# Numerical development—from cognitive functions to neural underpinnings

**DOI:** 10.3389/fpsyg.2014.01047

**Published:** 2014-09-19

**Authors:** Korbinian Moeller, Elise Klein, Karin Kucian, Klaus Willmes

**Affiliations:** ^1^Neurocognition Lab, Knowledge Media Research CenterTuebingen, Germany; ^2^Department of Psychology, University of TuebingenGermany; ^3^Section Neuropsychology, Department of Neurology, University Hospital Aachen, RWTH Aachen UniversityAachen, Germany; ^4^Center for MR-Research, University Children's Hospital ZurichZurich, Switzerland

**Keywords:** numerical development, approximate number system, developmental dyscalculia, mathematics learning disability, spatial-numerical association, language development

Living at the beginning of the 21st century requires being numerate because numerical abilities are not only essential for life prospects of individuals but also for economic interests of post-industrial knowledge societies (e.g., Butterworth et al., 2011). In recent years, numerical development has gained increasing research interest. Following this trend, we invited empirical and theoretical contributions for a Research Topic on *Numerical development—from cognitive functions to neural underpinnings*. We are grateful to all authors for their high-quality contributions, the reviewers for their constructive comments and suggestions in the interactive peer-review, and the publisher's editorial team for their excellent support.

The different contributions nicely illustrate that the construct *numerical development* does not denote a unitary, clearly circumscribed, and comprehensive entity. Instead, the empirical, review, opinion, and commentary articles clearly suggest that it is important to consider different empirical and theoretical perspectives evaluating cross-domain (e.g., language or spatial abilities) but also domain-specific [e.g., basic numerical competencies, approximate number system (ANS), spatial-numerical associations (SNA)] determinants of and influences on typical but also atypical numerical development.

A first set of studies investigated cross-domain as well as domain-specific influences on ***typical numerical development***. With respect to cross-domain influences LeFevre et al. (2013) showed a reliable impact of children's spatial abilities on numerical skills. Additionally, Durkin et al. (2013) observed that language ability is a unique predictor of actual and future numerical achievement. Getting closer to the domain of numerical cognition, Lafay et al. (2013) found that finger counting may be useful but not necessary to develop accurate symbolic numerical competencies. As regards basic numerical precursor competencies, two studies investigated the influence of the ANS on numerical development. Mejias and Schiltz (2013) suggest that the ANS may be targeted by educational strategies as it seems to be associated with socio-economic status. Lonnemann et al. (2013) observed that children's addition performance is associated with different markers of the ANS during development. For secondary school children, Huber et al. (2013) found the relation of multiplication and division to be stronger for easier problems and more skilled (i.e., higher grade) students. Moreover, two fMRI studies investigated neural correlates of numerical development. Mussolin et al. (2013) observed differential developments of the contributions of the right and left intraparietal sulcus (IPS) to magnitude comparison over age, while Gullick and Wolford (2013) found a fronto-parietal shift of activation with age in general, but also specific effects on the lateralization of IPS involvement for processing negative numbers. Finally, Lambrechts et al. (2013) observed that processing the continuous quantities time and space seems resilient to healthy aging similar to numerosity.

With a particular focus on the determinants of ***atypical numerical processing*** in mathematics learning disability (MLD) or developmental dyscalculia (DD), Mazzocco et al. (2013) showed that specific basic whole number misconceptions reliably predict atypical performance on Grade 8 arithmetic tests. Furthermore, Chu et al. (2013) found that even though inacuity of the ANS is a reliable predictor of risk for MLD it may not be its primary source. Nevertheless, Landerl (2013) evaluated the influences of other basic numerical competencies on numerical development and observed that children with DD exhibited specific impairments. Importantly, however, Haase et al. (2014) concluded that subtypes of MLD may not only be associated with content-related deficits but also more general impairments of information processing should be considered. In line with this view, Van Viersen et al. (2013) suggested that evaluating eye-fixation behavior may provide interesting new insights into the mechanisms underlying DD. In their Opinion, Kaufmann et al. (2013) argue that MLD/DD is a heterogeneous disorder resulting from individual differences in development or function at neuroanatomical, neuropsychological, behavioral, and interactional levels. Finally, Käser et al. (2013) described the development and first successful evaluation of a multimodal and adaptive computer-based training program for children with DD.

Another set of studies investigated interactions in the ***processing of numbers and space***. With respect to SNA, Knops et al. (2013) observed a reversed operational momentum (OM) effect in children with their degree of attentional control predicting the propensity to exhibit the OM effect. Kucian et al. (2013) commented that the left–right associations underlying the OM effect may dependent on development, reflecting an interaction of visuo-spatial and attentional processes with number related skills which might account for the non-observation of an OM effect in children (with DD). Moreover, Link et al. (2014) observed that unbounded number line estimation may be a valuable tool for assessing primary school children's spatial representation of number magnitude in an unbiased manner. Goldman et al. (2013) argued that the development of an analog comparison process and the specific processing of end stimuli contribute to the emergence of the mental number line. In their review, Patro et al. (2014) suggested a taxonomy for the classification of SNA from infancy to late preschool years. On the other hand, another two studies addressed direct interactions between the processing of numerical and physical magnitude as reflected by the size congruity effect (Henik and Tzelgov, 1982). Leibovich et al. (2013) found that numerical and physical magnitudes are represented by different, yet interactive systems. Ben-Shalom et al. (2013) observed that even preschoolers were able to process number magnitude information automatically. Finally, unrelated to space, Gabriel et al. (2013) suggested that conceptual knowledge about ***fractions*** and procedural knowledge about how to manipulate them should be distinguished. Faulkenberry (2013) commented that the evaluation of solution strategies might be beneficial to differentiate procedural and conceptual knowledge.

Extending the focus on ***language influences*** Imbo et al. (2014) and Klein et al. (2013) specifically investigated influences of number word inversion on numerical development. They found that language, but not working memory capacity, predicted the number of inversion errors and conclude that inversion-related difficulties do not fade over time, respectively. Lopes-Silva et al. (2014) observed the more basic perceptual phonemic awareness to predict number transcoding reliably, whereas magnitude processing and working memory did not.

As documented by this broad range of studies dealing with different aspects of numerical development—from behavioral performance to underlying neural substrates, from cross-sectional to longitudinal evaluations, from healthy to clinical populations—the current Research Topic brought together the expertise of researchers from different backgrounds and clearly advanced our understanding of numerical development—a topic with both scientific and every-day relevance.

## Conflict of interest statement

The authors declare that the research was conducted in the absence of any commercial or financial relationships that could be construed as a potential conflict of interest.
